# **Observation** of T-2 Toxin and HT-2 Toxin Glucosides from *Fusarium* *sporotrichioides* by Liquid Chromatography Coupled to Tandem Mass Spectrometry (LC-MS/MS)

**DOI:** 10.3390/toxins3121554

**Published:** 2011-12-20

**Authors:** Mark Busman, Stephen M. Poling, Chris M. Maragos

**Affiliations:** U.S. Agricultural Research Service, National Center for Agricultural Utilization Department of Agriculture Research, Peoria, IL 61604, USA; Email: Stephen.Poling@ars.usda.gov (S.M.P.); Chris.Maragos@ars.usda.gov (C.M.M.)

**Keywords:** *Fusariumsporotrichioides*, glucoside, trichothecene, wheat, oats, mass spectrometry

## Abstract

The trichothecenes produced by solid and liquid cultures of *Fusarium sporotrichioides* were evaluated with high performance liquid chromatography-tandem mass spectrometry (LC-MS/MS). Along with the expected T-2 toxin HT-2 toxin and neosolaniol, two additional compounds were detected, which had ions 162 *m/z* higher than those in the mass spectra of T-2 toxin or HT-2 toxin. Fragmentation behavior of these two compounds was similar to that of T-2 toxin and HT-2 toxin. Based on LC-MS/MS behavior, it is proposed that the two compounds are T-2 toxin 3-*O*-glucoside and HT-2 toxin 3-*O*-glucoside. Production of the two glucosides was measured in kernels from wheat and oat inoculated with *F. sporotrichiodes*, as well as in cultures grown in liquid media and on cracked corn or rice. Production of glucosides in wheat and oats suggest that they may also be present in naturally contaminated cereals.

## 1. Introduction

**Figure 1:**
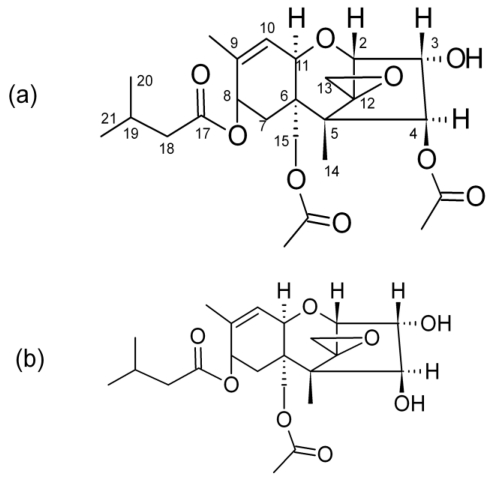
.Chemical structure of (**a**) T-2 toxin (m.w. 466.5) and (**b**) HT-2 toxin (m.w. 424.5).

T-2 toxin (Figure 1a) is one of a group of trichothecene mycotoxins produced by various species of Fusaria, including *Fusarium sporotrichioides*. These fungi are routinely found on commodities such as wheat, maize, oats, barley, and rice. A related compound, HT-2 toxin (Figure 1b), is thought to be produced by the deacetylation of T-2 toxin by microflora [1]. The toxicological effects of T-2 toxin and HT-2 toxin have been summarized in reports by the Joint Food and Agricultural Organization/World Health Organization (FAO/WHO) Expert Committee on Food Additives and the Council for Agricultural Science and Technology [2,3]. T-2 toxin is a potent inhibitor of DNA, RNA and protein synthesis, and shows immunomodulatory and cytotoxic effects both *in vivo* and *in vitro* [4]. The toxicities of T-2 and HT-2 toxins are quite similar [2]. *In vitro* human experiments have shown that T-2 toxin is rapidly metabolized to HT-2 toxin and, consequently, the toxicity of T-2 toxin *in vivo* might partly be attributed to HT-2 toxin [5].

There is a need to develop sensitive and accurate analytical methods for measuring these mycotoxins in cereals and cereal-based products in order to properly assess the risk of human exposure [6]. *Fusarium* trichothecenes are normally classified into two types: type-B trichothecenes all have a ketone functionality at the 8-carbon position, while the type-A trichothecenes have other types of functionalities (e.g., hydroxyl or ester) at the C-8 position. Gas-chromatographic (GC) and liquid-chromatographic (LC) methods coupled with mass spectrometry (MS), have been used for the analysis of type-A trichothecenes including T-2 toxin and HT-2 toxin [7]. High-performance liquid chromatography (HPLC) with UV detection is generally not applicable to sensitive detection of type-A trichothecenes due to the lack of a strong chromophore group within their structures [8]. HPLC methods for T-2 toxin and HT-2 toxin have been developed using derivatizing reagents and detection by fluorescence [6].

There have been reports of type B trichothecenes and zearalenone being “hidden” or “masked” by chemical modifications, particularly by reactions with sugars [9] that make them less likely to be detected using standard methods. Such reactions do not necessarily eliminate concerns over a material’s toxicity [10]. Instead, the toxin’s chemical identity is changed by modification so that the extreme specificity of a chemical assay will cause the modified toxin to be overlooked. Often, the reactions producing these “masked” toxins are reversible, allowing the toxin to be regenerated during evaluation with bioassays, or upon consumption by humans or livestock [11,12]. 

Chemical analysis of grain products for mycotoxins will likely not detect the “masked” toxins. However, animal or cell based assays or other assays based upon immunological response will often cross-react with these modified chemical species [13]. This cross reactivity in assay response is based upon the similarity of the structure of the modified species to its parent structure, or is a consequence of the regeneration of the parent compound during the course of the assay. Due to the differences in response to the hidden toxins, many have noted that chemical analysis is likely to under report the presence of toxins, as compared to levels detected in terms of biological or immunological means [13,14].

Berthiller, *et al.* have characterized a “masked” form of deoxynivalenol (DON) in wheat, deoxynivalenol 3-*O*-glucoside, and methods for its detection with high performance liquid chromatography-tandem mass spectrometric (LC-MS/MS) have been described [15].

The similarity in structures between DON and T-2 toxin suggests the trichothecene glucosidesmay also be formed by Fusarium sporotrichioides cultures. Here we describe the LC-MS/MS characterization of a putative T-2 toxin glucoside in *F. sporotrichioides* cultures and in grain from *F. sporotrichioides-*inoculated wheat and oats. We believe this is the first report of T-2 toxin or HT-2 toxin glucosides. 

## 2. Materials and Methods

### 2.1. Reagents

Except where noted otherwise, ultrapure water from a Millipore Milli-Q system (Millipore, Bedford, MA, USA) was used in the preparation of all reagents. T-2 toxin, HT-2 toxin, and neosolaniol were purchased from Sigma (St. Louis, MO, USA), and deoxynivalenol-3-glucoside, from Biopure (Tulln, Austria). Acetonitrile, ethyl acetate, methanol (HPLC grade), and other chemicals were purchased from Sigma. Cracked maize was purchased from Kelly Seeds (Peoria, IL, USA). White rice and mung beans were purchased from a local grocery store (Peoria, IL, USA). 

### 2.2. Fungal Isolates

*F. sporotrichioides* isolates NRRL-3299 and NRRL-3510 lyophilized on silica were obtained from the fungal collection at the National Center for Agricultural Utilization Research, Peoria, IL. The isolates were initially grown upon plates of V-8 juice agar. 

### 2.3. Fungal Culture in Liquid Media

GYEP (5% glucose, 0.1% yeast extract, 0.1% peptone) liquid medium was used for toxin production in 300 mL Erlenmeyer flasks. A small plug of inoculants was placed in 100 mL of GYEP medium. Liquid culture flasks were shaken continuously at 200 rpm on a Psycrotherm Model G-27 incubator (New Brunswick Scientific, Edison, NJ, USA). The flasks of culture in GYEP liquid media were maintained in the dark at 15 °C for 3 weeks. Cultures were harvested by filtration on a paper filter (2V, 125 mm, Whatman Paper Limited, Kent, England). Filtered fungal mass was extracted with ethyl acetate.

### 2.4. Fungal Culture on Cracked Corn or Rice Media

Cracked corn or rice was utilized as a support for toxin production. 50 g portions of the grain substrate were placed in 300 mL Erlenmeyer flasks with 11 mL of water and autoclaved for 30 min. Small plugs containing growing *F. sporotrichioides* were cut from the V-8 agar plate and placed on the substrate with an additional 11 mL of water and shaken vigorously for 30 seconds. The flasks were shaken for 30 seconds daily for the first three days. The flasks of culture on solid substrate were maintained in the dark at 15 °C for a total of 3 weeks. 

### 2.5. Extraction of Fungal Cultures on Cracked Corn or Rice Media

Upon completion of the culture period, flasks were extracted twice with 120 mL ethyl acetate. The extracts were combined and passed through filter paper (2 V, 125 mm, Whatman). The extracts for each strain were pooled and stored at room temperature in an amber glass container. 2 mL portions of the extract utilized for analysis were evaporated under a stream of nitrogen at ambient temperature, and resolubilized with an equal volume of methanol.

### 2.6. Comparison of Extraction Methods of Fungal Cultures on Cracked Corn

Upon completion of the culture period, inoculated cracked corn was divided into 25 g portions and placed into 125 mL flasks. Portions were extracted with 40 mL of either 1/1 (v/v) acetonitrile/water, 1/1 (v/v) methanol/water, 86/14 (v/v) acetonitrile/water, acetonitrile, methanol or ethyl acetate. The extracts were passed through filter paper (2 V, 125 mm, Whatman). 86/14 (v/v) acetonitrile/water, acetonitrile, methanol or ethyl acetate extracts were dried under a stream of nitrogen and resolubilized in 1/1 methanol/water. Extracts were subjected to analysis by LC-MS/MS.

### 2.7. Fungal Growth on Growing Wheat or Oats

Wheat (“Norm” cultivar) and oats (“Rodeo” cultivar) were grown for 4 weeks at 15 °C in 18-cm plastic pots of a standard pasteurized potting mix in a controlled environment chamber using a 14/10 light dark cycle. For the remaining growth time, the wheat and oats were grown in a greenhouse at 23 °C, with a 14/10 light dark cycle maintained by supplemental lighting. *F. sporotrichioides* isolates grown upon V-8 agar plates were suspended in mung bean medium, as described by Desjardins [16]. The suspension was diluted to produce a suspension of ~1 × 10^6^ spores per mL of mung bean medium. Suspensions of the medium were injected into wheat heads at anthesis. For oat heads, a hand sprayer was used to thoroughly wet the heads with inoculum. Upon inoculation, heads were covered with a plastic bag for three days. Wheat and oats were harvested upon maturity (approximately 120 days after planting) and hand threshed.

### 2.8. Extraction of Wheat or Oats for Toxin Analysis

Threshed wheat was weighed and ground with a Stein M2 laboratory grain mill (Steinlite Corporation, Atchison, KS, USA). Grain samples were extracted for toxin content. 10 g portions of ground grain were extracted with 20 mL of 86/14 (v/v) acetonitrile-water and shaken for 3 h. Extracts were filtered using a Whatman 5 V paper filter. 2 mL portions of the extracts were evaporated under a stream of nitrogen at 40 °C. The extracts were resolubilized with 2 mL of 1/1 methanol/water.

### 2.9. Flow Injection and High Perfomance Liquid Chromatography (HPLC)-Mass Spectrometry

Analyses were done by using a 1100 Series HPLC chromatographic system (Agilent Technologies, Waldbronn, Germany) interfaced to a QTRAP 3200 (Applied Biosystems/MSD Sciex, Foster City, CA, USA) equipped with a TurboV interface. MS interface conditions were as follows: nebulization temperature, 325 °C; curtain gas (nitrogen), 20 psi; nebulizer gas (nitrogen), 10 psi; auxiliary gas (nitrogen), 30 psi. The nitrogen flow for the source and collision gases was taken from the boil-off from a liquid nitrogen tank. For APCI experiments, a corona current of +1 μA was used. For ESI experiments a +4.5 kV potential was applied to the emitter. It was assumed that the tandem-MS behavior of analytes would be similar in experiments utilizing ESI-flow injection-MS/MS and APCI-LC-MS/MS modes of operation.

For flow injection experiments, the nebulization assisted electrospray ionization (ESI) mode of operation was utilized. Into a 0.3 mL min^−1^ flow of 1:1 acetonitrile/water containing 5 mM ammonium acetate, 10 μL plugs of analyte were inserted utilizing the injector valve integral to the mass spectrometer. 

Chromatographic conditions were as follows: injection volume: 5 μL; analytical column, Phenyl-Hexyl Luna (150 mm × 3.0 mm, 5 μm) (Phenomenex, Torrance, CA, USA); binary gradient, with the initial composition of the mobile phase, 30% acetonitrile: 70% water containing 5 mM ammonium acetate, kept constant for 5 min, then the acetonitrile content was linearly increased to 85% in 10 min, and kept constant for 10 min; flow rate of the mobile phase, 0.3 mL min^−1^. Analyses were conducted using atmospheric pressure chemical ionization (APCI) mode of operation. For LC-MS/MS evaluations of toxins the following transitions were used: T-2 toxin (484-305), HT-2 toxin (442-263), neosolaniol (400-305), T-2 toxin-glucoside (646-263), HT-2 toxin-glucoside (604-323). For chromatographic and MS/MS behavior the LC-MS/MS method was optimized with the use of solvent standards of T-2 toxin, HT-2 toxin and neosolaniol.

Levels of analytes were determined from integrated chromatographic peak areas for characteristic fragment ions obtained from collision induced dissociation of the toxin molecule adducted to an ammonium ion. Quantitation of T-2 toxin, HT-2 toxin and neosolaniol was done by comparison to peak areas obtained from toxin standard solutions.

## 3. Results and Discussion

### 3.1. Fungal Production of T-2 Toxin and HT-2 Toxin

*F. sporotrichioides* NRRL-3299 and NRRL-3510 were grown on cracked corn and extracted. Production of T-2 toxin and HT-2 toxin by the *F. sporotrichioides* isolates was confirmed by HPLC-MS/MS analysis using APCI. Multiple reaction monitoring experiments were performed to determine amounts of the toxins extracted from the fungal cultures. For both the NRRL-3299 and NRRL-3510, the concentrations of T-2 toxin and HT-2 toxin in the extract were 0.8 and 0.3 mg/mL, respectively. 

### 3.2. Product Ion MS/MS for T-2 Toxin and HT-2 Toxin

**Figure 2:**
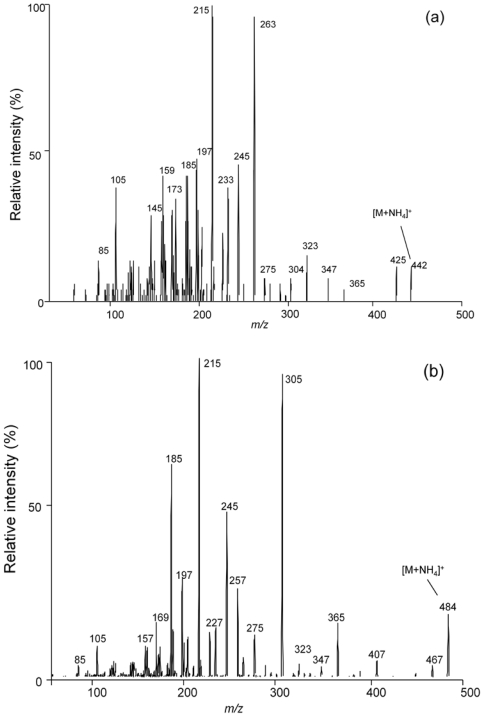
Product ion scan spectra (APCI, ion trap mode, scan range 100-1000 *m/z*) for the [M + NH_4_]^+^ ions of the (**a**) HT-2 toxin (*m/z* 442) and (**b**) T-2 toxin (*m/z* 484).

Product ion scan experiments (APCI, ion trap mode, scan range 100-1000 *m/z*) were performed to determine the fragmentation pattern for the [M + NH_4_]^+^ ions of the T-2 toxin and HT-2 toxin. (Figure 2) The fragmentation patterns obtained are extremely similar to those shown earlier by Kostiainen using a chemical ionization-MS/MS [17]. While Kostiainen postulated the structural basis for the larger fragments in the spectra, major smaller fragments were not interpreted and may represent results of major rearrangements of the ion during the dissociation. For example, in T-2 the *m/z* 215 product ion was postulated to result from the successive loss of neutral isovaleric acid, two acetic acid and formaldehyde molecules, while in HT-2 toxin the *m/z* 215 product ion was postulated to result from the successive loss of neutral isovaleric acid, acetic acid, formaldehyde and water molecules. However, even the lower mass fragments correspond closely with those obtained previously with very different instrumentation [17]. 

### 3.3. Parent Ion MS/MS for T-2 and HT-2 Related Compounds

**Figure 3:**
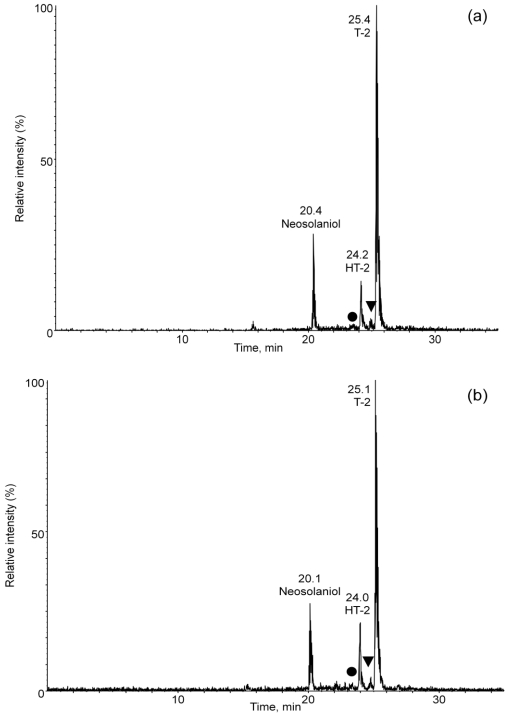
Parent ion chromatograms (ESI mode) for the (**a**) 185 and (**b**) 215 *m/z* ions selected for probing extracts for “masked toxins”.

Two intense low mass ions (185, 215 *m/z*) found in both the T-2 toxin and HT-2 toxin product ion spectra were selected for use in a parent ion scan experiment probing the extracts for “masked toxins” with ESI. In these experiments the first quadrupole (Q1) was scanned from 350 to 800 *m/z* while holding the third quadrupole (Q3) at one of the selected product ion masses. The chromatograms for experiments with an extract from isolate NRRL-3299 grown on cracked corn are shown in Figure 3. A peak in Figure 3a (labeled-▼) represents a parent ion 162 *m/z* higher (*m/z* 646) than that seen for the [M + NH_4_]^+^ ion of T-2 toxin. Likewise, a peak in Figure 3a (labeled-**●**) represents a parent ion 162 *m/z* higher (*m/z* 604) than that seen for the [M + NH_4_]^+^ ion of HT-2 toxin. Similarly, peaks in Figure 3b (labeled-▼ and-**●** representing parents of *m/z* of 646 and 604, respectively, are observed. These masses are those that would be expected for glucosides of T-2 toxin and HT-2 toxin. Similar results are seen for an extract from NRRL-3510 grown on cracked corn.

### 3.4. Production MS/MS for T-2 and HT-2

**Figure 4:**
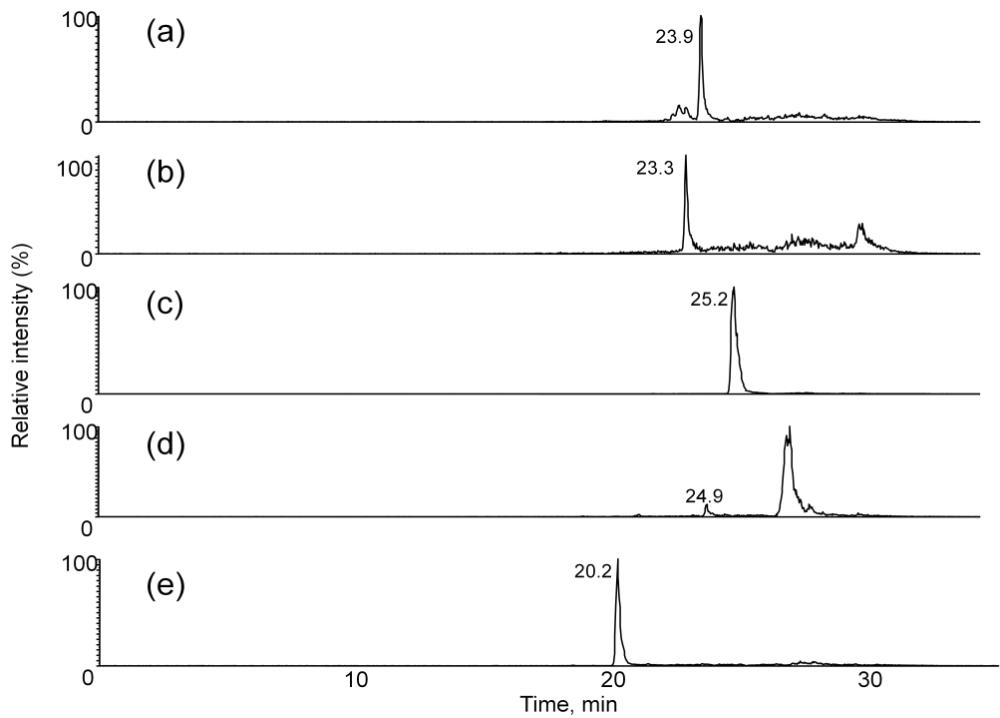
Extracted ion chromatograms for *m/z* (**a**) 442, (**b**) 604, (**c**) 484, (**d**) 646 and (**e**) 400 from full scan experiments (ESI, Q3 scan mode, scan range 100-1000 *m/z*) of the extracts were performed to evaluate the presence of [M + NH_4_]^+^ ions for T-2 toxin and HT-2 toxin, and their glucosides.

Full scan experiments (ESI, Q3 scan mode, scan range 100-1000 *m/z*) of the extracts were performed to evaluate the presence of [M + NH_4_]^+^ ions for T-2 toxin and HT-2 toxin, along with their glucosides. Figure 4 shows extracted ion chromatograms for *m/z* 442, 604, 484, 646 and 400 from an extract from NRRL-3299. These chromatograms represent those for masses expected for HT-2 toxin (23.9 min), HT-2 toxin-(glucoside) (23.3 min), T-2 toxin (25.2 min), T-2 toxin-(glucoside) (24.9) and neosolaniol (20.2), respectively. The large peak at ~26 min in the extracted ion chromatogram for *m/z* 646 (Figure 4d) was not identified.

**Figure 5:**
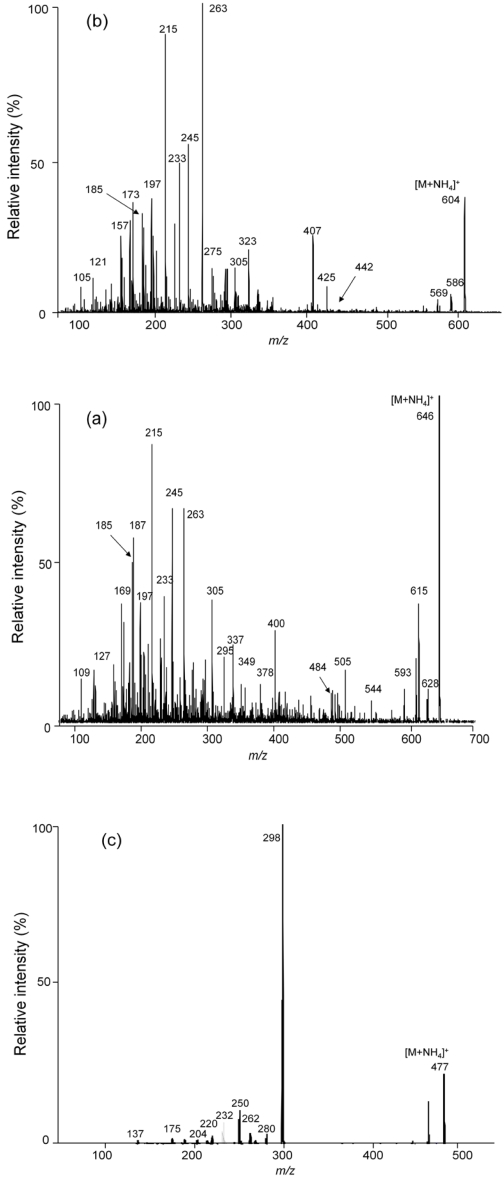
Product ion scan (ESI mode) for the (**a**) 646 and (**b**) 604 *m/z* ions from the NRRL-3299 *F. sporotrichioides* extract, along with a purified concentration standard of (**c**) DON-3-glucoside.

Flow injection ESI product ion scan experiments were conducted to determine fragmentation behavior for the 604 (HT-2 toxin-glucoside) and 646 (T-2 toxin-glucoside) *m/z* ions. Figure 5a,b shows product ion spectra for the two species. Small peaks in each spectrum are seen for the loss of 162 *m/z*. Major peaks in each spectrum correlate well with the spectra from the corresponding T-2 toxin and HT-2 toxin spectra. This indicates a behavior similar to that noted by Kostiainen, who observed the tendency of trichothecenes to successively lose substituents on the ring systems upon collisional activiation [17]. For comparison, Figure 5c shows product ion spectra for DON-3-glucoside ([M + NH_4_]^+^, *m/z* 477) under similar conditions. Kostiainen earlier noted that DON did not provide the extensive fragmentation seen in T-2 toxin and HT-2 toxin [17]. So, an analogous matching of the larger fragments and the major smaller fragments in the DON and DON-3-glucoside product ion spectra may not be reasonable.

**Figure 6:**
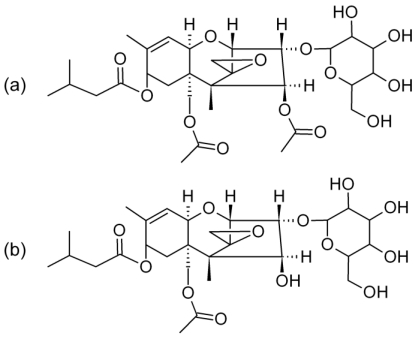
.Proposed chemical structures for (**a**) T-2 toxin (m.w. 628.7) and (**b**) HT-2 toxin (m.w. 586.6) glucosides.

Initial reports of the glucoside form of DON were accompanied by efforts to determine the position of attachment of the glucoside. Krska *et al*. described the chemical synthesis of the 3- and 15-glucoside forms. Comparison of the tandem MS behavior of the two molecules made it apparent that the naturally occurring form was the 3-glucoside [18]. Here, with the T-2 toxin, the only likely site of attachment of the glucoside is at the C-3 position, the location of the only free hydroxyl functionality. Therefore, we propose that the two additional compounds detected in *F. sporotrichioides* culture material are T-2 toxin-(3-*O*-glucoside) and HT-2 toxin-(3-*O*-glucoside) (Figure 6). However, the HT-2 toxin has an additional free hydroxyl functionality at the C-4 position, making that position an alternative location for the glucoside. 

### 3.5. LC-MS/MS of Glucosides and Extraction Efficiency Comparisons

**Figure 7:**
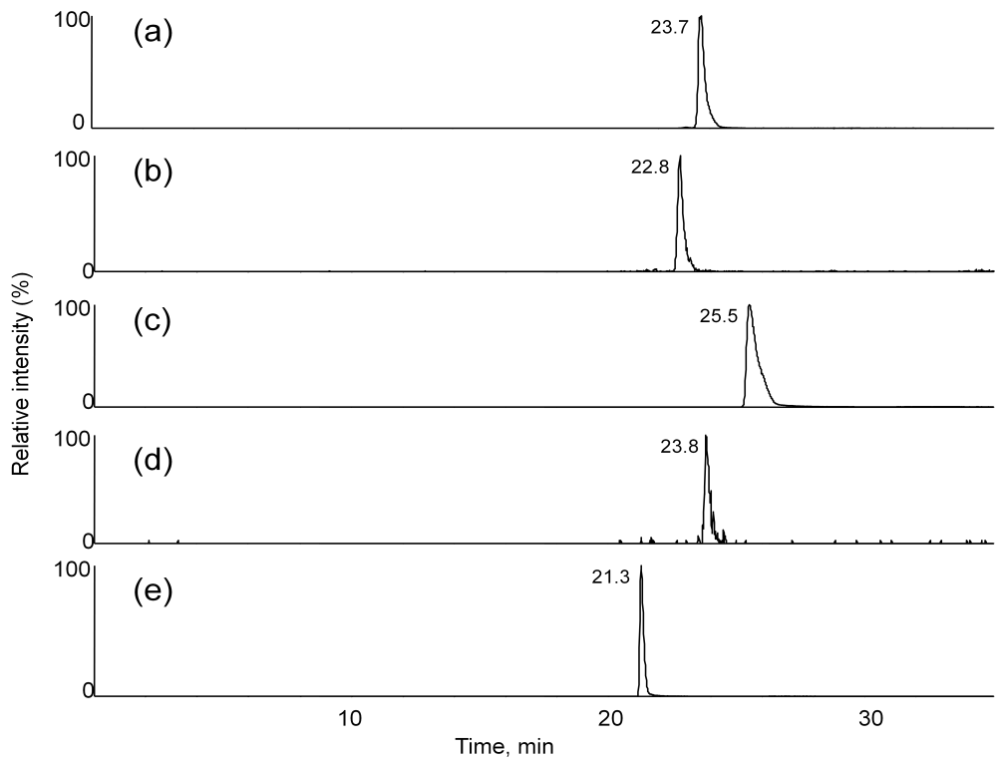
Selected ion chromatograms (ESI mode) for *m/z* (**a**) 442-263, (**b**) 604-323, (**c**) 484-305, (**d**) 646-263 and (**e**) 400-305 transitions from MS/MS experiments (selected reaction monitoring mode) of an extract were performed to evaluate the presence of [M + NH_4_]^+^ ions for HT-2 toxin, HT-2 toxin-glucoside, T-2 toxin, T-2 toxin-glucoside and neosolaniol in an extract from NRRL-3299.

A LC-MS/MS method was developed to screen for the presence of T-2 toxin, HT-2 toxin, T-2 toxin-glucoside, HT-2 toxin-glucoside and neosolaniol. The LC-MS/MS method was based upon observed characteristic fragments in the MS/MS spectra from each of the analytes. A LC-MS/MS chromatogram for an ethyl acetate extract from a *F. sporotrichioides* culture on cracked corn is shown in Figure 7. Absolute quantitative behavior of the method could not be verified without the presence of purified standards for the glucoside forms of the toxins. To evaluate the ability of several solvent systems to effectively extract both the T-2 toxin and HT-2 toxin, as well as their glucosides, portions from a single large scale *F. sporotrichioides* culture on cracked corn were extracted. Example results of LC-MS/MS evaluation of the extracts are summarized in Table 1. In this table the integrated area of the LC-MS/MS chromatographic peak area for each analyte in each analysis is normalized to the integrated peak area for T-2 toxin. This allows the comparison of the relative ability of analytes to be effectively extracted by each of the solvents. Although it was expected that the glucosides would be more hydrophylic than their unmodified analogues, the T-2 toxin glucosides were better extracted with ethyl acetate.

**Table 1:** Relative detection intensity comparisons for T-2 toxin, HT-2 toxin, neosolaniol, T-2 toxin-(3-glucoside) and HT-2 toxin-(3-glucoside) from cracked corn cultures. For each solvent system integrated ESI-LC-MS/MS chromatographic peak areas for compounds are normalized to the peak area for T-2 toxin.

**Solvent system**	**T-2 toxin**	**HT-2 toxin**	**Neosolaniol**	**T-2 toxin-glucoside**	**HT-2 toxin-glucoside**
Acetonitrile- water (1/1)	100	9.7	12.0	0	0.4
Acetonitrile- water (86/14)	100	8.7	11.2	0	0.5
Acetonitrile	100	9.6	11.5	0	0.5
Methanol	100	10.3	11.8	0	0.5
Ethyl acetate	100	14.3	18.2	0.048	0.5

### 3.6. Production of T-2 Toxin and HT-2 Toxin, along with the Proposed Glucosides with a Variety of Growth Conditions

The production of the glucosides of the toxins was evaluated on cracked corn and rice kernels and in GYEP liquid growth media. Further, production of the toxins was evaluated in growing heads of wheat and oats. Results of LC-MS/MS evaluations of the experiments are summarized in Table 2 for NRRL-3299. Similar results were observed for NRRL-3510 (data not shown). It is clear that significant levels of the T-2 toxin, HT-2 toxin and at least the HT-2 toxin-glucoside are found in every growth condition. Production of a neosolaniol glucoside was not noted utilizing the methods described.

**Table 2:** Production of T-2 toxin, HT-2 toxin, neosolaniol T-2 toxin-(3-glucoside) and HT-2 toxin-(3-glucoside) on cracked maize and rice kernels, growing wheat and oat heads, and in GYEP liquid medium. Detected ESI-LC-MS/MS signals for compounds are normalized to the detected signal for T-2 toxin.

**Support**	**T-2 toxin**	**HT-2 toxin**	**Neosolaniol**	**T-2 toxin-glucoside**	**HT-2 toxin-glucoside**
Cracked corn	100	14.3	18.2	0.048	0.5
Rice kernels	100	4.7	14.2	0.002	0.2
Growing wheat	100	43.5	0.0	0	19.2
Growing oats	100	11.1	0.0	0	1.9
GYEP-liquid medium	100	0.9	11.5	0	0.1

The T-2 toxin and or HT-2 toxin glucosides were detected in cultures grown on solid or liquid media. This suggests that the glucosides are produced by the fungi. This is in contrast to other glucoside forms of mycotoxins, which have been shown to be products of plant glucosyl tranferases [15]. However, the relatively higher levels the HT-2 toxin-glucoside in artificially inoculated wheat suggests that plant enzymes may also contribute to the glucoside formation.

## 4. Conclusions

While the investigation of “hidden” mycotoxins has progressed in recent years, it appears that more of these compounds still remain to be characterized. LC-MS/MS can be used for both structural characterization and quantification of these compounds. In the present work, we evaluated the formation of glucoside forms of T-2 toxin and HT-2 toxin by LC-MS/MS. In particular, fragmentation behavior during tandem mass spectrometry experiments was utilized to postulate the structures for these new compounds. To the best of our knowledge, this is the first report of type A trichothecene glucosides. Utilization of alternative analytical methods (synthetic standards, nuclear magnetic resonance, *etc*.) will facilitate the further characterization of these new compounds. Furthermore, production of the two compounds was characterized by means of LC-MS/MS in wheat and oat kernels, as well as in culture in liquid media and on solid support. Detection of these glucoside forms in artificially inoculated wheat and oats indicates that they may be present in naturally contaminated cereals. 
